# Selective Scale-Aware Network for Traffic Density Estimation and Congestion Detection in ITS

**DOI:** 10.3390/s25030766

**Published:** 2025-01-27

**Authors:** Cheng Jian, Chenxi Lin, Xiaojian Hu, Jian Lu

**Affiliations:** 1 Nanjing Les Information Technology Co., Ltd., Nanjing 210014, China; chengjianpaper@163.com; 2 Jiangsu Province Collaborative Innovation Center of Modern Urban Traffic Technologies, Southeast University, Nanjing 211189, China; huxiaojian@seu.edu.cn (X.H.); lujian_1972@seu.edu.cn (J.L.); 3 National Demonstration Center for Experimental Road and Traffic Engineering Education, Nanjing 211189, China; 4 School of Transportation, Southeast University, Nanjing 211189, China

**Keywords:** traffic congestion detection, convolutional neural networks, optical flow, video analysis

## Abstract

Traffic congestion detection in surveillance video is crucial for road traffic condition monitoring and improving traffic operation efficiency. Currently, traffic congestion is often characterized through traffic density, which is obtained by detecting vehicles or using holistic mapping methods. However, these traditional methods are not effective in dealing with the vehicle scale variation in surveillance video. This prompts us to explore density-map-based traffic density detection methods. Considering the dynamic characteristics of traffic flow, relying solely on the spatial feature of traffic density is overly limiting. To address these limitations, we propose a multi-task framework that simultaneously estimates traffic density and dynamic traffic congestion. Specifically, we firstly propose a Selective Scale-Aware Network (SSANet) to generate a traffic density map. Secondly, we directly generate a static congestion level from a traffic density map through a linear layer, which can characterize the spatial occupancy of traffic congestion in each frame. In order to further describe dynamic congestion, we simultaneously consider the dynamic characteristics of traffic flow, using the overall traffic flow velocity integrated with static congestion estimation for a dynamic assessment of congestion. On the collected dataset, our method achieves state-of-the-art results on both congestion detection and density estimation task. SSANet also obtains 99.21% accuracy on the UCSD traffic flow classification dataset, which outperforms other state-of-the-art methods.

## 1. Introduction

The process of urbanization has led to a rise in traffic congestion, which is gradually becoming a major source of stress in densely populated cities  [1]. Traffic congestion can lead to an increase in fuel consumption, resulting in elevated emissions levels and subsequent air pollution. Moreover, traffic congestion poses significant safety risks, such as higher rates of accidents and longer emergency response times. Hence, it is crucial to recognize traffic congestion promptly and undertake appropriate measures to minimize the damage caused by it [1].

The number of urban surveillance cameras has increased significantly in recent years, offering considerable potential for monitoring and providing more precise traffic information. Consequently, numerous researchers have focused on developing a vision-based congestion detection system to detect traffic congestion from these available surveillance videos [2]. A real-time congestion detection system can help transportation authorities alleviate congestion by allocating resources more effectively, such as adjusting signal timing or rerouting traffic.

Vision-based traffic congestion detection methods can be divided into detection-based microscopic methods and holistic analysis methods. Detection-based methods [3,4] determine traffic activity from motion features extracted from individual vehicle detection. However, the vehicles in surveillance videos exhibit significant scale variations and mutual occlusion, rendering detection-based methods inadequate for accurate vehicle localization. Traditional holistic analysis methods [5,6,7,8,9] analyze the texture feature of the whole image to extract the spatial information and regress into several discrete congestion levels or continuous congestion factors. These holistic methods avoid detecting individual vehicles from video and usually contain better real-time performance [5]. However, the lack of localization for individual vehicles poses limitations when dealing with rare traffic scenarios.

In recent years, density-map-based methods have been widely used for vehicle counting, making them an effective approach for representing the spatial distribution of dense vehicles [10]. Since it effectively handles dense traffic scenarios, we consider estimating traffic congestion from vehicle density maps.

In this paper, we proposed a density-map-based holistic dynamic method to analyze traffic congestion. In contrast to detection-based methods, the proposed density-map-based approach does not detect motion state of individual vehicles, hence avoiding potential tracking errors in congested traffic scenarios. Considering traffic surveillance videos have high occlusion and strong perspective effects, we proposed a Selective Scale Local Self-attention (SSLSA) to encode multi-scale dense vehicles. When dealing with objects that have significant scale differences, it is crucial to establish connections between query tokens and the surrounding objects. To achieve this goal, SSLSA carries out the self-attention within multiple windows centered around the query token and selectively aggregates these local attention features to generate multi-scale local attention. Meanwhile, the optical flow algorithm is introduced to detect traffic velocity, which would help to obtain holistic motion feature of traffic flow and estimate dynamic congestion factor from spatio-temporal domain.

In short, the contributions of this paper are summarized as follows:

(1) A novel framework is proposed for estimating traffic density and congestion level from surveillance videos.
(2) Selective Scale-Aware Network (SSANet) is developed to generate the vehicle density map and estimate end-to-end static congestion factor. SSANet is equipped with the Selective Scale Local Self-Attention (SSLSA) mechanism in its encoder layers, which can effectively hand with scale variation.
(3) A novel holistic traffic flow velocity estimation method is proposed, by utilizing the density map to guide optical flow map analysis.

The remaining part of this paper is as follows: Section 2 reviews previous works; Section 3 elaborates the principles and implement of the proposed method; Section 4 introduce the dataset and discuss the experiments results; Section 5 is the conclusion and the future research.

## 2. Related Work

### 2.1. Local Attention in Vision Transformers

This section provides a comparative analysis of the technical aspects of our proposed method against existing advanced attention mechanisms. The Swin Transformer [11] introduces a hierarchical architecture that captures multi-scale features through a novel shifted window approach for self-attention, significantly enhancing computational efficiency while maintaining robustness in image processing tasks. CSwin Transformer [12] enhances the traditional Swin Transformer [11] by introducing a cross-shaped window attention mechanism, which improves the model’s ability to capture both local and long-range dependencies in a more efficient manner. This innovation not only reduces the computational cost but also boosts performance across various vision tasks. NAT [13] introduces a novel attention mechanism that focuses on local neighborhoods within feature maps, enhancing the model’s ability to capture spatial dependencies and contextual information effectively. CMT [14] analyzes the importance of local information in Vision Transformers by integrating locality directly into the transformer architecture, which facilitates better feature extraction from small regions of images. Recognizing the sparsity and local feature of the shallow self-attention module, DilateFormer [15] captures hierarchical features using several windows with dilated grids.

The aforementioned works [11,12,13,14] focused on the locality of self-attention while neglecting the importance of features from different levels of granularity. Although other studies [15,16] have considered multi-scale representations, they have overlooked the significance of dynamic fusion. In this paper, to effectively address the multi-resolution features of vehicles in traffic monitoring videos, we propose a novel Selective Scale Local Self-Attention (SSLSA) method.

### 2.2. Congestion Detection Method

The present studies focused on congestion detection methods can be broadly categorized into two groups. The first detection-based methods involves the collection and analysis of traffic flow parameters, such as traffic volume, space headway, vehicle speed and traffic density, which analyze the motion of vehicles from a microscopic perspective. The second category adopts a holistic approach by analyzing the texture features of traffic images from a macroscopic perspective to classify traffic images.

Detection-based methods typically integrate with computer vision algorithms, such as K-means clustering algorithm [1], Kanade-Lucas-Tomasi (KLT) algorithm [17], and Optical Flow algorithm [18]. Gao et al. [5] directly estimated traffic congestion by integrating a traffic parameter layer into Faster-RCNN [19]. Hu et al. [20] proposed an algorithm that classifies congestion videos based on the isolation of moving vehicles. Ke et al. [4] proposes a multi-dimensional approach for detecting traffic congestion using a fusion of visual features and convolutional neural networks. Ribeiro et al. [3] proposed a traffic flow classification methods to recognize traffic congestion, classifying traffic activity in three steps: vehicle monitoring, feature extraction, and classification.

The holistic classification methods are generally based on direct feature extraction and classification. Wang et al. [6] proposed a locality constraint distance metric learning to detect traffic congestion. Luo et al. [7] investigated methods for classifying traffic conditions in low-frame videos. Pamula et al. [8] classified traffic images to detect congestion levels according to traffic density. Gao et al. [5] obtained a congestion factor by considering both road occupancy and traffic density, and subsequently, analyzed the holistic traffic image to estimate the congestion factor. Lin et al. [9] proposed a congestion detection framework based on the image classification method. They introduced a spatial attention module to address the variability of different scenes. Corresponding efforts have been made to prevent the direct detection and tracking of individual vehicle within dense traffic flow. However, challenging traffic scenarios still pose limitations to the aforementioned methods. Moreover, incorporating physical laws into machine learning models [21,22] for congestion detection is also a promising area of research. By integrating domain-specific knowledge, such as traffic flow theory and physical constraints, machine learning models can potentially achieve better generalization and robustness in complex traffic scenarios.

## 3. Method

**Figure 1:**
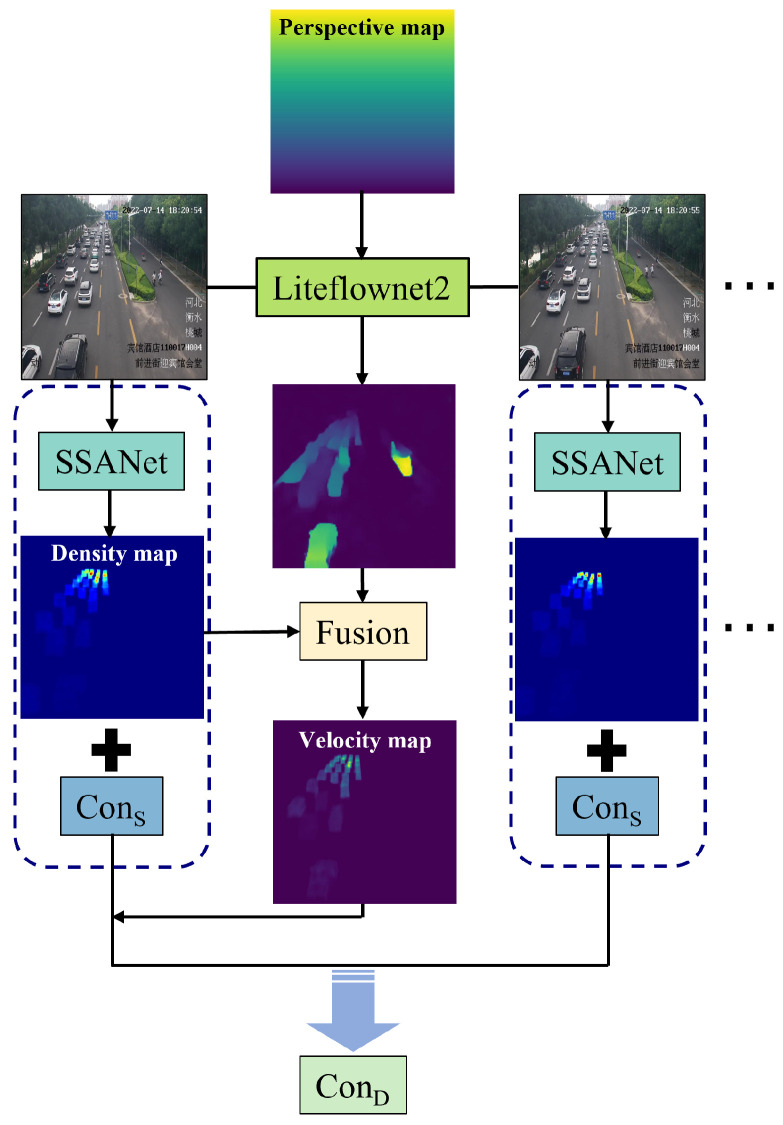
The work pipeline of the proposed density-map-based congestion detection method. SSANet takes video frames as input, generates a density map, and takes the end-to-end static congestion level $Con_{S}$ as output. Meanwhile, the optical flow map between two frames is estimated by the optical flow algorithm. Element-wise multiplication of the optical flow map and density map results in the flow density map, from which the average traffic flow speed can be estimated. Finally, the traffic velocity is weighted to $Con_{S}$ and averaged to obtain the dynamic congestion factor $Con_{D}$ of the input frame sequence.

In this paper, we estimate the multi-dimensional dynamic congestion factor from temporal and spatial dimensions. The dynamic congestion factor consists of a static congestion factor which is related to the density feature and the occlusion feature as well as the average traffic velocity. As shown in Figure 1, we proposed a spatio-temporal network to estimate the dynamic congestion status. The proposed SSANet model is used for density map generation and the estimation of static congestion factors. Note that the static congestion factor is determined by two key spatial characteristics of traffic flow: traffic density and spatial occupancy. The optical flow algorithm is applied to the analysis input frame sequence and extracts the traffic velocity, which is used to estimate traffic congestion from the temporal domain.

### 3.1. Static Congestion Quantification

In this paper, a method which utilizes multi-dimensional visual feature is proposed to describe traffic congestion. Specifically, the dynamic motion feature and static spatial feature are combined to analyze traffic conditions from both the temporal and spatial domains. Since the possibility of traffic congestion is greater under the condition of high traffic density, high road occupancy, and low traffic velocity [4], we describe the congestion status based on these traffic features.

Traffic density reflects the relationship between vehicles and road occupancy reflects the relationship between vehicles and overall road [4]. In the process of traffic status detection, we notice that road occupancy and traffic density increasing significantly when traffic becomes congested. Considering both of these two static spatial features can be obtained from an individual frame, we propose a static congestion factor $Con_{S}$ which related to the density feature and occupancy feature to estimate the congestion from the spatial domain. $Con_{S}$ can be expressed as follows:

(1) $$Con_{S}=\omega_{\sigma }\sigma +\omega_{k}k,$$

where σ denotes the occupancy feature and *k* denotes the density feature. Furthermore, we propose an end-to-end convolution neural network SSANet to directly estimate $Con_{S}$ for inputting frames sequences. The process of the proposed method is shown in Figure 1.

The parameters of Equation (1) serve to constrain the effect of the corresponding features. Therefore, it is crucial to ascertain the correct weight to accurately estimate and detect traffic congestion. To this end, our model necessitates the appropriate normalization of features of varying dimensions in order to infer correct weights for evaluating the visual features of each dimension. Specifically, we establish the ideal range of occupancy feature σ to be within the interval [0, 1], and accordingly, set the weight $\omega_{\sigma }=1$. Since the number of vehicles accommodated in each scene is different, we identify different density weight $w_{k}$ for different scenes. Specifically, we determine the density in the most congested traffic flow as $k_{max}$ and free traffic flow as $k_{min}$. Given the direct correlation between traffic density and the perceptible performance of road congestion as inferred from previous analyses, we assign density weight $\omega_{k}=1/\left(k_{max}-k_{min}\right)$. The detailed explanation of ground truth static congestion factor generation can be found in Section 3.5.2.

### 3.2. Structure of the Selective Scale-Aware Network

**Figure 2:**
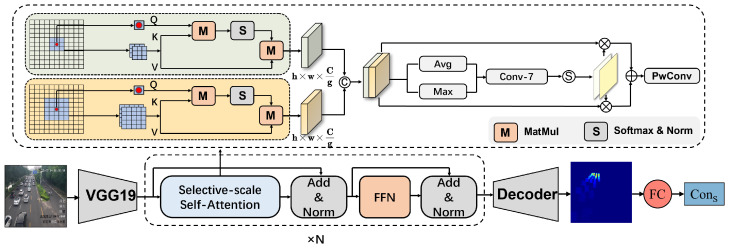
Structure of the Selective Scale-Aware Network (SSANet). In each window branch, local self-attention is performed around the red query block within different window scales (the default window size is 3×3, 5×5). Finally, these multi-scale local features are concatenated and input to the dynamic aggregation module for adaptive aggregation.

In this section, we give a introduction to the network structure of SSANet. As shown in Figure 2, SSANet is mainly composed of a backbone network, several cascaded encoder layers, and a CNN-based decoder network.

#### 3.2.1. Backbone

The Encoder network adopts the first 19 layers of VGG19 to extract the primary feature map as embedding, comprising 16 convolutional layers and 3 max-pooling layers. The configuration of the backbone follows the settings described in the original paper. VGG19 is pre-trained on the ImageNet dataset to expedite the optimization of the training process. The input image with the size of H×W is processed by VGG19 to the size of $\frac{H}{16}\times \frac{W}{16}$. Then, the feature map is upsampled to $\frac{H}{8}\times \frac{W}{8}$ and fed into the encoder layers.

#### 3.2.2. Encoder Layer

Each encoder layer consists of two main components: a Selective Scale Local Self-Attention mechanism (SSLSA) and a feedforward network (FFN). The SSLSA is calculated, followed by a residual connection and a layer normalization:

(2) $$X^{\prime }=\mathrm{LayerNorm}\left(X+\mathrm{SSLSA}\left(X\right)\right).$$

Then, FFN is applied independently to each position in the sequence:

(3) $$FFN\left(X^{\prime }\right)=\mathrm{ReLU}\left(X^{\prime }W_{1}+b_{1}\right)W_{2}+b_{2},$$

where $W_{1}$ and $W_{2}$ are weight matrices and $b_{1}$ and $b_{2}$ are biases. After passing through the feedforward network, another residual connection is applied, followed by layer normalization:

(4) $$X^{\prime \prime }=\mathrm{LayerNorm}\left(X^{\prime }+FFN\left(X^{\prime }\right)\right).$$

#### 3.2.3. Selective Scale Local Self-Attention

In this paper, to achieve effective perception of multi-scale vehicles in congested traffic scenarios, we propose a Selective Scale Local Self-Attention mechanism (SSLSA). Based on Multi-Head Self-Attention (MHSA) [23], we first define a local self-attention mechanism. Specifically, we compute self-attention for each query token by selecting keys and values within a local window of size l×l. This mechanism aims to capture local dependencies more effectively while reducing computational complexity compared to global self-attention.

The input feature map is represented as $X\in R^{h\times w\times d}$, where *h* and *w* represent the height and width of the feature map, respectively, and *d* is the dimensionality of the feature vectors. For each position (i,j) in the feature map, we can define a local window of size l×l to focus on the spatial features surrounding that position. The query $q_{\left(i,j\right)}$, key $k_{\left(i,j\right)}$, and value $v_{\left(i,j\right)}$ representations at each spatial location can be computed as follows:

(5) $$Q_{\left(i,j\right)}=X_{\left(i,j\right)}W^{Q},\;K_{l}=X_{\left(m,n\right)}W^{K},\;V_{l}=K_{\left(m,n\right)}W^{V},$$

where m∈[i−l/2,i+l/2],n∈[j−l/2,j+l/2], the weight matrices $W^{Q}$, $W^{K}$, and $W^{V}$ are essential components of the local self-attention mechanism, enabling the transformation of input features into query, key, and value representations, where $W^{Q},W^{K},W^{V}\in R^{d\times d_{k}}$, *d* is the dimensionality of the input feature vectors, and $d_{k}$ is the dimensionality of the query vectors. The local self-attention for the spatial position (i,j) is subsequently calculated as:

(6) $$\mathrm{Attention}\left(Q_{\left(i,j\right)},K_{l},V_{l}\right)=\mathrm{softmax}\left(\frac{Q_{\left(i,j\right)}K_{l}^{T}}{\sqrt{d_{k}}}\right)V_{l}.$$

The local self-attention mechanism allows each spatial position to derive its outputs based on the interactions with nearby position.

Then, two local self-attention branches with different window sizes are employed to capture multi-scale features, which are then concatenated as:

(7) $$X_{l}=\mathrm{Attention}\left(Q_{\left(i,j\right)},K_{l},V_{l},l\right),l=\left\{3,5\right\},$$

(8) $$U=\mathrm{CAT}\{X_{3},X_{5}\},$$

where $X_{l}\in R^{h\times w\times \frac{C}{2}}$ and CAT{·} indicates concatenation of feature maps along the channel dimension. Considering the difference in receptive fields between feature maps, a selective dynamic method is adopted to enhance feature fusion. First, we utilize average pooling operation $P_{avg}\left(\cdot \right)$ and maximum pooling operation $P_{max}\left(\cdot \right)$ to generate spatial feature descriptors for *U*:

(9) $$X_{avg}=P_{avg}\left(U\right),X_{max}=P_{max}\left(U\right).$$

Then, $X_{avg}$ and $X_{max}$ are concatenated and processed by a 7×7 convolution $F_{7\times 7}^{2\to 2}\left(\cdot \right)$ to generate the attention map with size of h×w×2. The sigmoid function is then applied to generate the weight masks:

(10) $$W_{SA}=\sigma \left(F_{7\times 7}^{2\to 2}\left(CAT\left\{X_{avg},X_{max}\right\}\right)\right),$$

where σ(·) represents the sigmoid function. Each attention branch is multiplied with the corresponding dynamic spatial mask and concatenated. Finally, a 1×1 convolutional layer $F_{1\times 1}^{C\to C}\left(\cdot \right)$ is set to achieve feature refinement and fusion:

(11) $$\hat{X}=F_{1\times 1}^{C\to C}\left(\mathrm{CAT}\left\{W_{SA}\left[0\right]⊗X_{3},W_{SA}\left[1\right]⊗X_{5}\right\}\right).$$

#### 3.2.4. Decoder Network

The decoder network is responsible for the dimensionality reduction of the feature map and the generation of the output density map. The detailed structure is presented in Table 1. The size of the output density map is $\frac{H}{8}\times \frac{W}{8}$, which can display the spatial distribution of the vehicle and count the object by summing.

### 3.3. Traffic Flow Velocity Estimation

It is noted that the static congestion level generated by SSANet belong to the static traffic analysis. Considering traffic may be flowing freely when the static congestion level is large in special cases, it is necessary to take dynamic traffic analysis into account when estimating congestion. In this paper, in order to address this issue, we propose a density-map-guided traffic flow velocity estimation algorithm. Firstly, we introduce optical flow algorithm (such as Liteflownet2 [24]), a lightweight model for optical flow estimation. Then, we show how to eliminate the influence of perspective effects on velocity estimation through perspective transformation. Finally, we introduce how to guide the optical flow field through a density map to better estimate traffic flow velocity.

#### 3.3.1. Optical Flow Estimation

In this paper, we choose LiteFlowNet2 [24] to generate optical flow information for its lightweight architecture and real-time performance. The efficiently inference of LiteFlowNet2 can ensure the real-time performance of traffic congestion detection, and the low computational requirement is beneficial for further edge deployment. Nonetheless, any lightweight optical flow estimation algorithm can be employed in our framework.

#### 3.3.2. Density-Map-Guided Traffic Velocity Estimation

**Figure 3:**
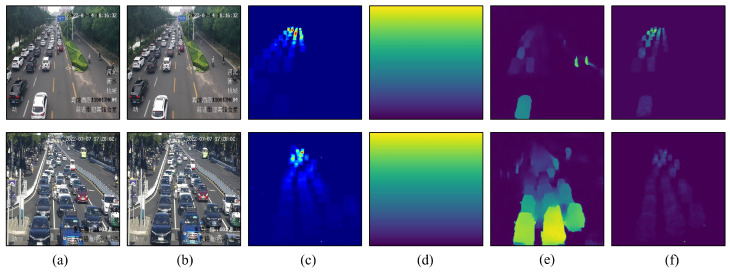
(**a**) Video frame in *t*; (**b**) Video frame in t+1; (**c**) Estimated density map of video frame in *t*; (**d**) Perspective map; (**e**) Optical flow map; (**f**) Flow density map.

Upon receiving the optical flow map as a result, the subsequent task is to derive the traffic flow motion details from it. The optical flow map comprises a (H,W,2) matrix that stores the motion information of each pixel along both the x-axis and y-axis. For the convenience of calculation, we use $\Delta d=\sqrt{{\left(\Delta x\right)}^{2}+{\left(\Delta y\right)}^{2}}$ to calculate the displacement of each pixel and generate the optical flow map (as shown in Figure 3e).

However, directly using optical flow maps to measure traffic flow speed leads to two challenges. The perspective effect results in that the size and pixel displacement of distant vehicles would be smaller than that of the vehicles closer to the camera, thus heavily influencing the traffic flow velocity. On the other hand, determining the displacement of each individual vehicle rather than each pixel from optic flow maps is not intuitively feasible. In this regard, we propose a two-stage strategy to tackle this predicament: *(i)* augmenting the importance of vehicle displacement from the upper part of the image (that is, from farther away from the camera) via the perspective map; *(ii)* utilizing a density map to supervise the optical flow field by decreasing the interior of each vehicle pixel displacement. These steps are explained in detail below.

In order to account for the potential impact of perspective transformation on traffic flow velocity estimation, we use a perspective map (as shown in Figure 3d) to incorporate this transformation into the optical flow generation. Specifically, since distant vehicles move fewer pixels at the same speed, we adjust the weighting in the upper region of the optical flow image to reflect this reality, placing greater emphasis on those pixels representing vehicles situated farther from the camera. To achieve this, following the methods in [6], we base the weighting of pixels in the upper region of images on the width of the road depicted in the images, which is similarly impacted by perspective transformation. Our approach successfully mitigates the perspective effect in traffic flow velocity, as demonstrated in our results.

After eliminating the perspective effect, the next step is to investigate how to extract the motion information of vehicles from the optic flow map, which represents the displacement of each pixel. The density map represents the probability of vehicle presence in the 2D spatial domain. Considering this, we proposed a simple yet effective method that involves multiplying the density map (Figure 3c) and the optical flow map (Figure 3e) point by point to utilize the density map to dilute the displacement of each pixel. In this way, we obtained the probability distribution of vehicle displacement in the spatial domain, which is called a velocity map (Figure 3f). Similar to the integration of a specific area of the density map to obtain the vehicle count, integrating over a certain area of the flow density map can provide the displacement of all vehicles within that area. The resulting optical flow density map projects the displacement of each vehicle in the image.

### 3.4. Dynamic Traffic Congestion Detection

#### 3.4.1. Dynamic Congestion Quantification

For dynamic motion features, we propose to analyze the adjacent frames through optical flow algorithm to obtain the traffic flow velocity $v^{*}$. Next, let us consider how to characterize the effect of traffic flow velocity on traffic conditions. $1/\log\left(v^{*}\right)$ was used as a weight to represent the impact of low traffic flow velocity on congestion [4]. However, this weighting scheme is difficult to handle scenarios where $v^{*}=0$, denoting the occurrence of vehicles coming to a complete stop as a result of overwhelming congestion. We desire a function of $v^{*}$ with values ranging within (0, 2) and decreasing with increasing $v^{*}$ when $v^{*}>0$. In this way, for two scenarios with the same $Con_{S}$, the $Con_{D}$ decreases as the traffic flow velocity increases and increases as the traffic flow velocity decreases. By adopting the threshold value (0,2), we can assign higher $Con_{D}$ to slow-moving traffic flows without the risk of over-dispersing the spatial distribution due to varying velocity. As a result, we choose $a/(b+v^{*})$ as a weight function, where a>0, b>0. The proposed formula is given as follows:

(12) $$Con_{D}=\frac{1}{n-1}\sum \frac{a\times Con_{S}}{b+v^{*}},$$

where $Con_{D}$ denotes the dynamic congestion factor of one video clip which estimate form spatio-temporal dimension, $Con_{S}$ denotes the static congestion factor of each extracted frame which can be end-to-end estimated form spatio-temporal network, $v^{*}$ denotes the taffic flow velocity, *n* denotes number of extracted frames from the video clip, and the values of variables *a* and *b* are determined in Section 4.3.2. When presented with a 5-s video as input, the initial step involves extracting an average of 10 frames from it, resulting in the extraction of two frames per second. The selection of this specific frame rate is based on the trade-off between computational overhead and the generation of reliable optical flow maps by the optical flow generation model. A higher frame rate would increase the computational demands, while a lower frame rate would render the generation of a trustworthy optical flow map challenging. Moreover, analyzing the maximum of 25 frames per second is redundant, and would not lead to significantly higher accuracy. Thus, the use of two frames per second is deemed a more fitting sampling approach. The pipeline of the proposed spatio-temporal dynamic traffic congestion detection method is shown in Algorithm 1. **Algorithm 1:** Dynamic Congestion Detection.
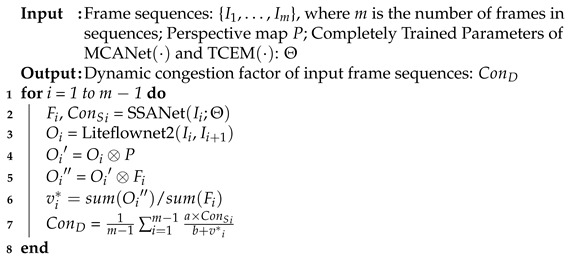


#### 3.4.2. Traffic Congestion Detaction

For the ith video frame, the density map $F_{i}$ and ${Con_{S}}_{i}$ can be generated from the MCANet:

(13) $$F_{i},{Con_{S}}_{i}=\mathrm{SSANet}\left(I_{i};\Theta \right).$$

Meanwhile, the optical flow map is generated between two input frames $I_{i}$ and $I_{i+1}$:

(14) $$O_{i}=\mathrm{Liteflownet}2\left(I_{i},I_{i+1}\right).$$

We also apply the post-processing of optical flow maps. Specifically, to handle noise and improve robustness, we apply bilateral filtering to the computed optical flow maps. This helps smooth noisy regions while preserving motion boundaries, which are crucial for accurate motion representation. The processed optical flow map is multiple by density map to eliminate the perspective effect:

(15) $${O_{i}}^{\prime }=O_{i}⊗P.$$

Then, the output optical flow map is multiplied by the density map to get the flow density map ${O_{i}}^{\prime }$:

(16) $${O_{i}}^{\prime \prime }=O_{i}^{\prime }⊗F_{i}.$$

The multiplication of the optical flow map with the density map can inherently suppresses the impact of erroneous flow vectors in areas with sparse or noisy input (e.g., poorly textured regions in low-light scenarios). This step ensures that only the displacement of vehicles is counted in the overall traffic flow displacement, effectively characterizing the speed of the overall traffic flow. Then, we can obtain the holistic displacement of the traffic flow by dividing the sum of all elements in ${O_{i}}^{\prime }$ by the sum of all elements in $F_{i}$:

(17) $$v_{i}^{*}=sum\left({O_{i}}^{\prime \prime }\right)/sum\left(F_{i}\right)$$

Finally, we obtain dynamic congestion factor $Con_{D}$ as follows:

(18) $$Con_{D}=\frac{1}{m-1}\sum_{i=1}^{m-1}\frac{a\times {{Con_{S}}_{i}}^{\prime }}{b+{v^{*}}_{i}}.$$

In this way, we can dynamically evaluate the traffic congestion situation of input traffic videos from the spatio-temporal domain. Thanks to the strategy of conducting static traffic congestion evaluation from density maps, the proposed method can better cope with vehicle occlusion and strong perspective effects in urban surveillance videos, thus achieving better congestion recognition results. In addition, we also introduce the optical flow algorithm and combine it with density maps to evaluate the holistic motion characteristics of traffic flow. Considering both the spatial and temporal factors of traffic flow, the proposed method can effectively evaluate traffic congestion.

### 3.5. Ground Truth Generation

#### 3.5.1. Ground Truth Density Map Generation

**Figure 4:**
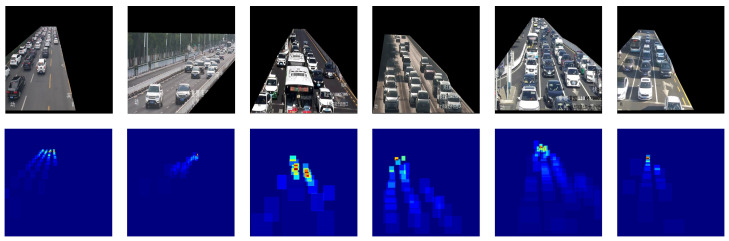
Some example video frames with ROI from the collected dataset.

As mentioned above, the vehicle is labeled by bounding box $B=(x_{1},y_{1},x_{2},y_{2})$, where $\left(x_{1},y_{1}\right)$ is the the coordinate of the left top point and $\left(x_{2},y_{2}\right)$ is the the coordinate of the right bottom point. The construction of the ground truth density map is executed through the utilization of bounding boxes. Specifically, the density value F(x) of pixel *x* is determined by the coverage of a given set of bounding boxes denoted as O(x):

(19) $$F\left(x\right)=\sum_{i=1}^{N}\frac{1}{A(o_{i})},$$

where $A(o_{i})$ denotes the area of the bounding box $o_{i}$ and *N* denotes the total number of bounding box. Density maps sequences generated in frame sequences from several case scenarios are shown in Figure 4.

#### 3.5.2. Ground Truth Static Congestion Factor Generation

In order to generate the determined static congestion factor $Con_{S}$, we should firstly obtain density feature σ and occlusion feature ω. In our approach, we define the density feature as the ground truth vehicle count in ROI, and is determined as the aggregate number of bounding boxes present in the dataset. For the occlusion feature, we discuss two fundamental methods for determining traffic occupancy [25]. The first technique utilizes pixel counting, while the second method involves area calculations. The pixel-count-based method calculates the number of foreground pixels while maintaining a constant road background, serving as an indicator of the proportion of the road that is occupied by foreground objects. However, this method highly dependent on the integrity of foreground detection and is not suitable for data formats without segmentation map labels. To address this limitation, Ke et al. [4] proposed an alternative approach that involves the computation of the area of the Minimum Enclosing Rectangle (MER) for each connected region of the foreground object. By utilizing a fixed road background, the MER area provides an estimation of the extent to which the foreground object occupies the road. This method can better solve the evaluation of road occupancy, and hence, we calculate the occupancy feature by calculating the total area of all label boxes as follows:

(20) $$\omega =\frac{U(A\left(o_{1}\right),A\left(o_{1}\right)...A\left(o_{N}\right))}{S},$$

where U() denotes the union, A(o) denotes the area of the bounding box *o*, and *S* denotes the ROI area. Then, we can obtain static congestion factor $Con_{S}$ for each frame followed by Equation (1). In our method, an Encoder–Decoder–LSTM model is proposed to generate end-to-end $Con_{S}$.

## 4. Experiment

In this paper, SSANet is proposed for static congestion estimation. Considering the flow characteristics of the traffic, the LiteFlowNet2 optical flow model is introduced for traffic velocity estimation, which is weighted to the static congestion factor to estimate congestion dynamicly. Proposed models are developed under the Pytorch 1.8.1 framework using Python 3.8. Training and testing were carried out on computer with a GTX 3090ti GPU with 24 GB memory, an Intel i5-12600KF CPU, and 64 GB of RAM. The SGD (Stochastic Gradient Descent) was applied in training process. The learning rate was set to $1\times 10^{-7}$, the decay rate and momentum value were set to $2\times 10^{-5}$ and 0.9. Batch size and number of epochs were set to 6 and 400.

### 4.1. Traffic Congestion Detection Dataset Collection

In order to better meet the requirement of urban traffic control, we created a crowded vehicle counting dataset COTRS (Congestion Traffic Sense) dataset by carefully selecting fifteen cameras that covered a variety of locations, camera angles, and traffic conditions. The fifteen camera locations are primarily situated at intersections and along urban expressways. This strategic placement allows for the observation of various traffic conditions, including congestion scenarios typical of urban settings. Bounding boxes were used for annotating the videos with larger vehicle. By analyzing the traffic density in videos captured by widely distributed roadside cameras, it is easy to understand the current traffic conditions at a specific intersection or a shorter road segment.

### 4.2. Experiments for Static Congestion Estimation on COTRS

#### 4.2.1. Evaluation Metrics

The current study adopts both the mean absolute error (MAE) and mean square error (MSE) as evaluation metrics for assessing the performance of the proposed model. The MAE is employed to evaluate the precision of the model, whereas the MSE reflects the stability of model. The definitions of these indices are provided as follows:

(21) $$MAE=\frac{1}{N}\sum_{i=1}^{N}\left|C_{i}-C_{i}^{GT}\right|,$$

(22) $$MSE=\sqrt{\frac{1}{N}\sum_{i=1}^{N}{\left|C_{i}-C_{i}^{GT}\right|}^{2}},$$

where *N* denotes the number of images in one test sequence, $C_{i}$ denotes the predicted static congestion factor, and $C_{i}^{GT}$ denotes the static ground truth congestion factor.

#### 4.2.2. Comparison with Object Counting Methods

Our task bears certain resemblances to the vehicle counting task, though it differs in that the latter is concerned with producing a density map, which is summed to estimate traffic density, whereas our task aim to regress the generated density map to the static congestion factor. In this paper, in order to compare the effect of the proposed method, three baseline methods for object counting will also be used for traffic congestion estimation: CSRNet [26], CAN [27], and Repmobilenet [10].

**Figure 5:**
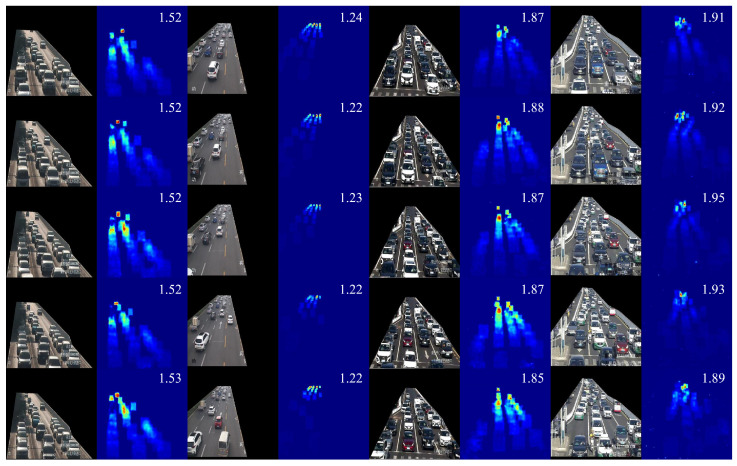
Density map sequences estimated from frame sequences of different traffic conditions in the TRCOS dataset. The number in the upper right corner of the density map denotes the estimated static congestion factor $Con_{S}$.

As shown in Table 2, SSANet achieves better performance than baseline models on the COTRS dataset. Compared with the best performance of the baseline model (FCN-rLSTM), SSANet reduces the MAE from 1 to 2. This evaluation results validate the effectiveness and robustness of proposed SSANet, since the testing data span a variety of congestion levels, camera perspectives, weather conditions, and times of day. Estimated density map sequences and static congestion factors of SSANet from four scenarios with dense traffic situations are shown in Figure 5. The density map learned by SSANet can provides accurate vehicle spatial locations without the need for foreground segmentation. Even in very crowded traffic situations, the proposed SSANet still can generate accurate high-quality density maps and yield accurate static congestion factor. In can be observed that the proposed method is effective for static congestion estimation and can effectively avoid the influence of shadows on the generation of density maps.

**Figure 6:**
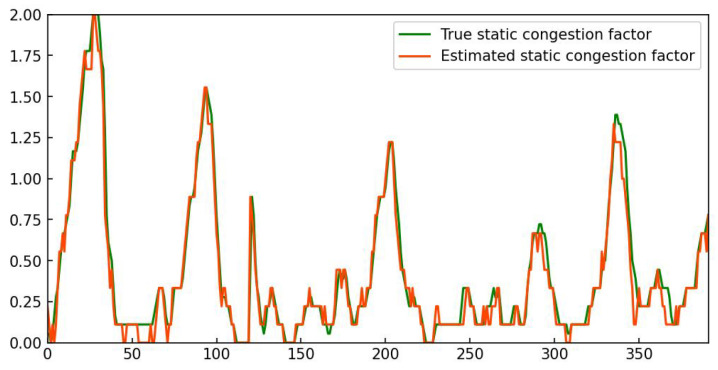
Comparison result between the estimated and ground truth $Con_{S}$ of one scene.

The comparison curve between the estimated and ground truth count of the static congestion factor is shown in Figure 6. From the counting curves, it can be observed that the proposed SSANet precisely estimate static congestion caught by several surveillance cameras over a long-term time series.

#### 4.2.3. Comparison with Attention Mechanisms

We also compared the proposed SSLSA with several advanced attention mechanisms: MHSA [23], Swin [11], and CSwin [12]. Specifically, several attention mechanisms are introduced to replace SSLSA, creating different model variants. As shown in Table 3, the model configured with SSLSA achieved the best performance, which may be attributed to SSLSA being able to effectively capture features at different spatial resolutions, allowing the model to better represent both fine-grained and coarse-grained information.

### 4.3. Experiments for Congestion Detection on COTRS

#### 4.3.1. Evaluation Metrics

When evaluating congestion detection method, the evaluation protocol includes various statistical metrics such as accuracy, precision, recall, and F-score. These metrics are calculated using the following formulas:

(23) $$\begin{array}{cc} \; & \mathrm{Accuracy}\;=\frac{{\mathrm{TP}}_{k}+{\mathrm{TN}}_{k}}{ALL} \end{array}$$

(24) $$\begin{array}{cc} \; & \;\mathrm{Precision}\;=\frac{1}{n}\sum_{k=1}^{n}\frac{{\mathrm{TP}}_{k}}{{\mathrm{TP}}_{k}+{\mathrm{FP}}_{k}} \end{array}$$

(25) $$\begin{array}{cc} \; & \;\mathrm{Recall}\;=\frac{1}{n}\sum_{k=1}^{n}\frac{{\mathrm{TP}}_{k}}{{\mathrm{TP}}_{k}+{\mathrm{FN}}_{k}} \end{array}$$

(26) $$\begin{array}{cc} \; & \;F\text{-}\mathrm{measure}\;=\frac{2\times \;\mathrm{Precision}\;\times \;\mathrm{Recall}\;}{\;\mathrm{Precision}\;+\;\mathrm{Recall}\;} \end{array}$$

where the *k*th state refer to congestion, slow, or free traffic states, ${TP}_{k}$ represents the number of videos correctly classified as being in the *k*th state, ${FP}_{k}$ indicates the number of videos that were misclassified as belonging to the kth state, ${TN}_{k}$ denotes the number of videos that were accurately classified as not belonging to the *k*th state, ${FN}_{k}$ represents the number of videos that were misclassified as not being in the *k*th state, $P_{k}$ denotes the number of videos that were accurately classified, and $P_{k}$ denotes the number of videos that were misclassified.

#### 4.3.2. Experimental Results

To determine the related parameters, we crawled the speed distribution of the road network in Hengshui for one week, with an average speed of 40 km/h and approximately 10 pixels per half frame. Using this velocity as a benchmark, we assigned a weight of less than 1 to traffic flows that operate at velocity above this benchmark, resulting in a further reduction in congestion factors. Conversely, we assigned a weight of greater than 1 to traffic flows that operate at velocity below this benchmark, leading to a further increase (no more than twice) in congestion factors. Hence, based on the weight function $a/(b+v^{*})$, we hope that when $v^{*}>10$ (average speed of the road network), the weight is less than 1; while when $v^{*}<10$, the weight is greater than 1. Therefore, we have identified a = 20 and b = 10.

**Figure 7:**
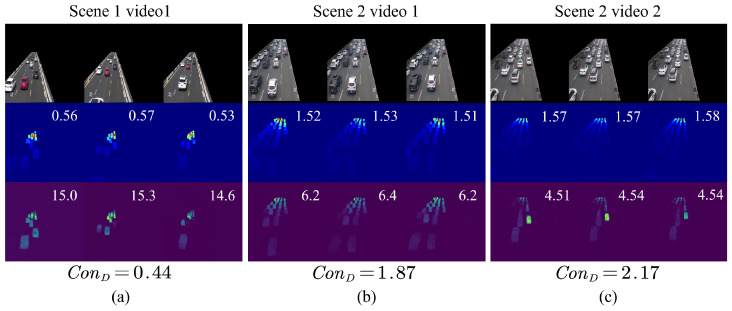
Density map sequences (middle row) and flow density map sequences (bottom row) estimated from our pipeline in TRCOS dataset. The number in the upper right corner of the density map denotes the estimated static congestion factor $Con_{S}$; the number in the upper right corner of the flow density map represents the estimated traffic flow velocity.

The original input images, density maps, and flow density maps in the two scenes are shown in Figure 7. The obtained $Con_{S}$ of scene1 video1 (Figure 7a) and scene2 video1 (Figure 7b) is 0.44 and 1.87, respectively, which means that the congestion status of the former is significantly lower than that of the latter at this point in time. In order to further illustrate the importance of dynamic congestion estimation, we selected two video clips with almost similar $Con_{S}$ but different traffic flow velocity for comparison. As shown in Figure 7, it can be observed that although the two scenarios have almost identical static congestion factors $Con_{S}$, their dynamic congestion factors $Con_{D}$ differ significantly due to differences in traffic flow motion feature. Therefore, a reasonable estimation of the congestion situation has been achieved.

**Figure 8:**
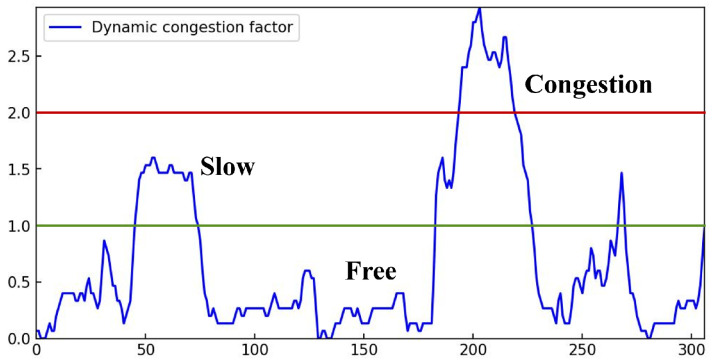
Comparison result between the estimated and ground truth $Con_{D}$.

By integrating a range of multi-dimensional traffic feature, including traffic density, traffic velocity, and road occupancy, our approach carries out the computation of a continuous measure of traffic congestion, denoted as $Con_{D}$. This enables us to assess traffic congestion from multiple perspectives, which we have expressed through the identification of three distinct states: congestion, slow, and free. Our model allows the adjustment of the upper and lower bounds for congestion detection, with $\varepsilon_{max}$ and $\varepsilon_{min}$ being defined as 2.0 and 1.0, respectively. As illustrated in Figure 8, the value of $Con_{D}$ exhibits changes that correspond to variations in road status and is closely linked to this variable.

In order to demonstrate the superiority of the proposed method, two congestion detection methods [3,4] were trained and tested on the collected dataset, using the ground truth vehicle box. For [3], the YOLOv2 and Faster-RCNN detectors were trained, and the Kalman filter and Deepsort algorithms were employed for vehicle tracking. The resulting outputs were then fed into a classifier for training to obtain a model for detecting traffic congestion. For [4], the YOLOv5+Deepsort algorithm was utilized for vehicle detection and tracking. The performance of the two methods on the dataset is presented in Table 4.

#### 4.3.3. Evaluation of Processing Speed

Due to the real-time requirement of traffic congestion recognition in the Intelligent Traffic System, the real-time test of traffic flow classification model is very important. On our platform, performing one SSANet inference only takes 0.009 s, and performing LiteFlowNet2 inference takes 0.03 s. It should be noted that we do not need to process all frames like methods based on vehicle detection and tracking, but only extract two frames every second, thus achieving superior real-time performance. The proposed pipeline processes a five-second video in 0.39 s, achieving good real-time performance.

### 4.4. Experiments on the UCSD Dataset

#### 4.4.1. UCSD Dataset

**Figure 9:**
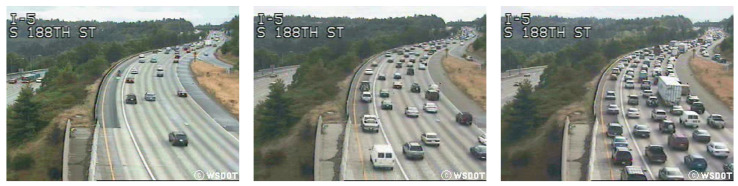
Frames extracted from videos contained in UCSD dataset showing different types of traffic flow status. (**Left**): *LIGHT*. (**Middle**): *MEDIUM*. (**Right**): *HEAVY*.

The UCSD dataset is a widely used benchmark in the field of traffic flow classification and analysis. This dataset consists of video sequences captured from a stationary camera positioned above a roadway. It includes diverse traffic scenarios, along with variations in traffic conditions, such as different times of day and varying levels of congestion. The dataset contains labeled video for various traffic flow classes, i.e., *LIGHT, MEDIUM*, and *HEAVY*, and records approximately 22 min of traffic footage, with each video featuring 10 fps and a total of 50 frames of size 320×240. The 254 videos in the UCSD dataset have been categorized into three traffic flow categories: 165 videos of light traffic, 45 videos of medium traffic, and 44 videos of heavy traffic. Figure 9 showcases an example image from each category in the UCSD dataset.

#### 4.4.2. Implementation and Training Process

**Figure 10:**
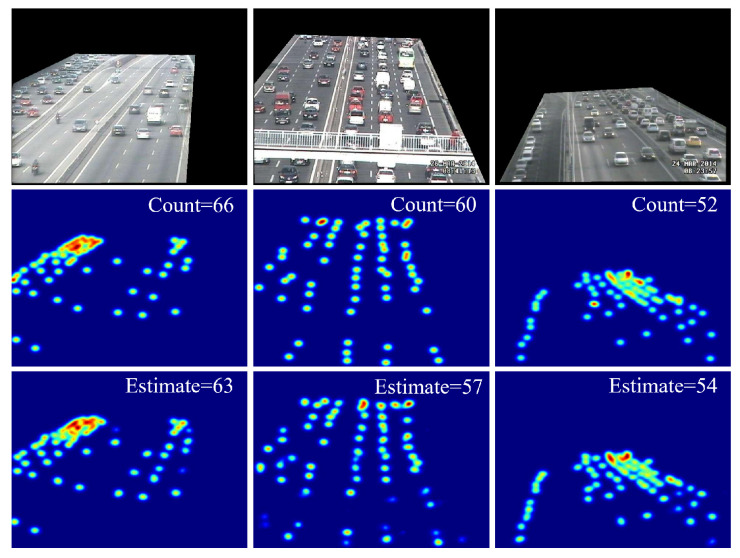
Density map estimated from multiple cameras in the TRANCOS dataset.

In light of the absence of any vehicle annotation information in the UCSD dataset, we were unable to produce end-to-end static congestion factor for frames of the UCSD dataset. Therefore, we approximate the static congestion factor with the density feature. In order to generate density maps and evaluate congestion, we introduced the TRANCOS vehicle counting dataset [28] for density map generation. TRANCOS is a publicly available dataset specifically designed for vehicle counting; it consists of 1244 images taken by surveillance cameras in a variety of heavy traffic situations. The dataset contains a total of 46,796 labeled vehicles and regions of interest (ROIs) of each scene for evaluation. Figure 10 illustrates several scenarios and the generated vehicle density maps from the TRANCOS dataset.

Experiments were conducted using the same setup as described in [9]. A total of 254 videos were divided into training and testing sets in a 3:1 ratio. A total of five experiments were conducted, and the final results presented are the averaged values from these five trials. In each experiment, for each video in the UCSD dataset, *T* frames $F_{1}$, ..., $F_{T}$ are sampled from the video and processed into tensors of size 640×480×3. *T* frames are combined to form a tensor of size 640×480×3×T; the proposed SSANet processes this tensor and gives the final prediction. For the number of input frames, T=5 was applied in each set of experiments.

#### 4.4.3. Experimental Results

**Figure 11:**
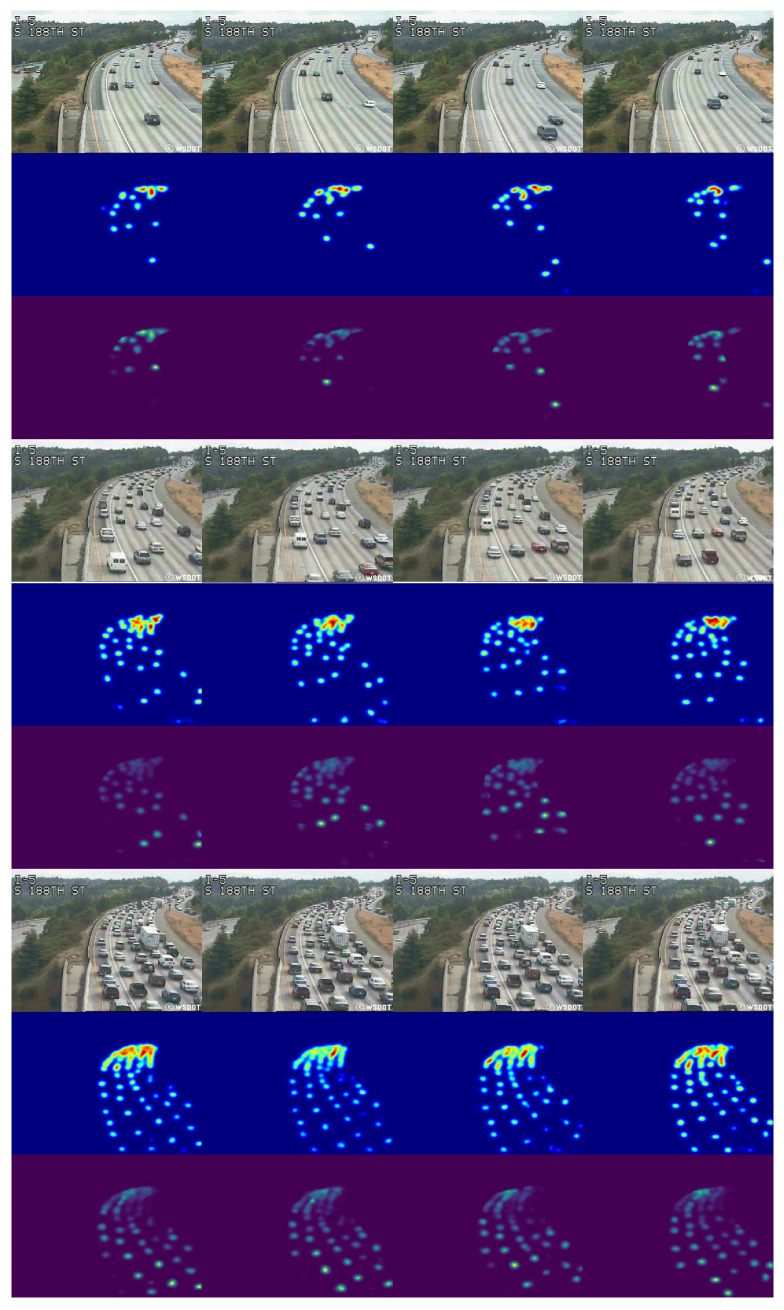
Density map sequences and flow density map sequences estimated from frame sequences of different traffic conditions in the UCSD dataset.

We compared our method with state-of-the-art approaches on the UCSD dataset. The comparison results are presented in Table 5, where our method achieved an accuracy of 99.21%, matching the performance of the SA-MobileNetV2 [9]. In comparison to the detection-oriented approach described in [3], our method based on density maps demonstrates superior recognition capability with regard to small-sized vehicles, and circumvents the issue of missed detections arising from a chain of post-processing methods, including NMS, when operating in congested traffic environments. Simultaneously, the introduction of motion information in our approach allows for precise classification of the traffic flow state MEDIUM, even in scenes with dense vehicles yet high flow velocity. Figure 11 presents the visualization of three traffic state detection scenarios. This result clearly shows that our method can help to obtain representative features of the traffic flow state.

## 5. Conclusions

In this paper, in order to overcome the effect of a strong perspective effect and high occlusion on congestion estimation, we proposed a deep spatio-temporal framework to generate sequences of density map and detecting traffic congestion from these density maps. We propose the utilization of the Selective Scale-Aware Network (SSANet) to learn the spatial distribution characteristics of traffic flow and the utilization of the optical flow algorithm to learn the motion features of traffic flow. SSANet achieves accurate feature representation and novel end-to-end trainable mapping from pixels to the static traffic congestion factor in the spatial domain. Meanwhile, the optical flow algorithm is applied to generate traffic flow velocity, which would help to generate the dynamic congestion level. To evaluate our algorithm, we collect and annotate a large-scale traffic video dataset which consists of 77,429 annotated vehicles in 6318 frames from 15 scenes. For the static congestion factor estimation task, SSANet achieves an MAE of 0.117 in the collected dataset, outperforming other baseline models. For the congestion detection task, the proposed method achieves 95.70% accuracy, outperforming other state-of-the-art methods, and it also has a processing speed far exceeding that of other methods. Our method also obtains 99.21% accuracy on the UCSD traffic flow classification dataset, outperforming the state-of-the-art methods. However, we have observed that the proposed model may miss some vehicles during nighttime vehicle detection, possibly due to misidentifying vehicles as background in low-light conditions. In future work, we aim to address these challenges by building a more comprehensive dataset that incorporates various adverse weather conditions and lighting scenarios. This will enable us to evaluate and adapt our methodology to these more complex environments. Additionally, we plan to explore advanced techniques, such as domain adaptation or data augmentation strategies, to artificially simulate harsh environmental conditions for training purposes. Techniques leveraging multi-modal data, including thermal imaging and radar sensors, may also be explored to complement visual data and improve detection performance in low-visibility situations.

## Figures and Tables

**Table 1:** Details of the decoder network.

Operation	Kernel Size	Stride	Filter Depth	Activation
Conv2D	3×3	1	256	ReLU
Conv2D	3×3	1	128	ReLU
Conv2D	1×1	1	1	-

**Table 2:** Estimation errors of the static congestion factor on the COTRS dataset.Note that the best results are highlighted in **bold**.

Method	MAE	MSE
CSRNet [26]	0.158	0.177
CAN [27]	0.148	0.147
Repmobilenet [10]	0.157	0.183
**SSANet (Ours)**	**0.117**	**0.158**

**Table 3:** The ablation analysis of different attention mechanisms.Note that the best results are highlighted in **bold**.

Method	MAE	MSE
Baseline	0.165	0.177
Baseline + MHSA [23]	0.142	0.154
Baseline + Swin [11]	0.132	0.157
Baseline + CSwin [12]	0.162	0.173
Baseline + SSLSA(ours)	**0.117**	**0.158**

**Table 4:** Comparison of congestion detection on the COTRS dataset. Note that the best results are highlighted in **bold**.

Method	Accuracy	Precision	Recall	F-Meature
Ke et al. [4]	0.9213	0.9169	0.9191	0.9180
Yolo2 + Kalman Filter [3]	0.8898	0.8772	0.8925	0.8848
Faster R-CNN + DeepSort [3]	0.9134	0.9001	0.9181	0.9090
SA-ResNet [9]	0.9134	0.9001	0.9181	0.9090
**SSANet**	**0.9606**	**0.9614**	**0.9581**	**0.9598**

**Table 5:** Comparison of congestion detection with the UCSD dataset. Note that the best results are highlighted in **bold**.

Method	Accuracy (%)
Chan and Vasconcelos [29]	95.24
Andrews Sobral et al. [30]	95.63
Derpanis and Wildes [31]	95.30
Asmaa et al. [32]	96.37
Luo et al. [7]	96.90
Luo et al. [33]	97.64
Yolo2 + Kalman Filter [3]	98.03
Faster R-CNN + DeepSort [3]	98.82
SA-MobileNetV2 [9]	**99.21**
**SSANet**	**99.21**

## Data Availability

The data presented in this study are available on request from the corresponding author due to the data restrictions from partners.
